# Modern chronic heart failure therapy in context of pulmonary banding to avoid heart transplantation

**DOI:** 10.1186/2194-7791-2-S1-A16

**Published:** 2015-07-01

**Authors:** S Recla, B Steinbrenner, T Logeswaran, L Rüblinger, D Schmidt, J Bauer, C Apitz, J Thul, D Schranz

**Affiliations:** Children's Cardiology Center at, Giessen University Hospital, Germany

## Background

Dilated cardiomyopathy (DCM) is a leading cause of cardiac death in children. Approximately 30% of children die or need cardiac transplantation in the first year after diagnosis. We established a protocol to improve the outcome in this high-risk population.

## Patients and methods

We present our experience in 21 patients (mean age 8 month, mean weight 6 kg) treated in our institution from 2006 to 2015. The patients were diagnosed with DCM with a highly impaired function of the LV (mean EF 17%) and a conserved function of the RV (mean EF 52%). Our protocol for medical enhancement of left ventricular recovery in association with pulmonary artery banding involves the use of a highly specific β_1_-blocker, an angiotensin-converting enzyme inhibitor and an aldosterone antagonist. Our therapy aims to reduce oxygen consumption and improve oxygen delivery. Heart rate control is the most important goal; therefore clonidine is given after surgical procedure and digoxin in long-term treatment if heart rate remains high despite adequate β_1_-blocker therapy. To improve oxygen delivery our goal is to archive haemoglobin levels of 12-14 g/dl, therefore Erythropoietin is given as long term treatment. Additional treatments include supplementation of carnitine, coenzyme Q, riboflavin or thiamine.

## Results

At a mean follow up of 36 month (range 2-120) freedom from death was 91% and freedom from heart transplantation was 85%. Surviving patients showed a significant improvement in left ventricular ejection fraction (from 17 ± 6 to 50 ± 11%) and LVEdD (z-score from +7 ± 2 to +1.7 ± 1.9). The levels of BNP improved significantly (from 3222 ± 2756 to 70 ± 56 pg/ml).

## Conclusion

Our data suggest that the medical and surgical approach described may result in a markedly improved medium-term outcome in children with DCM. Further studies are required to evaluate the long-term-outcome of these patients.

